# MiR-200c restoration inhibits FOXP3 and metastatic spread in breast cancer: evidence from in vitro and in vivo models

**DOI:** 10.1186/s12885-026-15574-6

**Published:** 2026-02-14

**Authors:** Nashwa El-Khazragy, Ahmed Alsolami, Ahmed M. Aref, Marwa N. M. Hassan, Mohamed S. Othman

**Affiliations:** 1 Department of Clinical Pathology-Hematology and Ain Shams Medical Research Institute (MASRI), Faculty of Medicine, Ain Shams University, Cairo, 11566 Egypt; 2 Department of Internal Medicine, College of Medicine, University of Ha’il, Ha’il, Saudi Arabia; 3 Faculty of Biotechnology, October University for Modern Sciences and Arts (MSA), Giza, Egypt; 4 Medical Biochemistry and Molecular Biology Department, Faculty of Medicine, Ain Shams University, Cairo, Egypt; 5 Biochemistry Department, College of Medicine, University of Ha’il, Ha’il, Saudi Arabia

**Keywords:** MicroRNA-200, Breast cancer, FOXP3 transcription factor, Neoplasm metastasis, Gene expression regulation, Neoplastic, Cell invasion

## Abstract

**Background:**

Metastatic breast cancer remains a leading cause of cancer-related mortality in women, often driven by molecular pathways that promote invasion and immune evasion. MicroRNA-200c (miR-200c) is a known tumor suppressor that inhibits epithelial-mesenchymal transition (EMT), while FOXP3, a transcription factor typically associated with regulatory T cells, is aberrantly expressed in breast cancer cells and may contribute to tumor progression. This study investigates whether targeting the miR-200c/FOXP3 axis can suppress metastasis in breast cancer.

**Methods:**

Metastatic (MDA-MB-361, MDA-MB-468) and non-metastatic (MCF-7) breast cancer cell lines were transfected with miR-200c mimic or inhibitor. Cell proliferation, apoptosis, and invasion were assessed using MTT, Annexin V/PI staining, and transwell assays. FOXP3 mRNA and protein levels were quantified using qRT-PCR and immunohistochemistry. A metastatic mouse model was established via intracardiac injection of tumor cells, followed by treatment with miR-200c mimic, inhibitor, or Cisplatin.

**Results:**

MiR-200c overexpression significantly suppressed proliferation and invasion and enhanced apoptosis in metastatic cells. FOXP3 mRNA and protein expression were downregulated in mimic-treated cells and tissues, while miR-200c inhibition led to increased FOXP3 expression. In vivo, miR-200c mimic treatment reduced tumor burden and metastatic infiltration in the brain and lungs. A strong inverse correlation between miR-200c and FOXP3 was observed (*r* = − 0.82, *p* < 0.01).

**Conclusion:**

MiR-200c restoration inhibits FOXP3 and suppresses metastatic progression in breast cancer. Targeting the miR-200c/FOXP3 axis presents a novel and promising therapeutic approach for advanced breast cancer.

## Background

Breast cancer remains one of the most prevalent malignancies and the leading cause of cancer-related deaths among women worldwide, with over 2.3 million new cases diagnosed annually [1–3]. Despite significant advances in early detection, targeted therapy, and adjuvant treatment, a considerable number of breast cancer patients develop recurrent or metastatic disease, which is largely incurable and associated with poor prognosis [4, 5]. Among the key challenges in managing advanced breast cancer is the emergence of molecular subtypes and pathways that drive resistance, immune evasion, and aggressive metastatic behavior [6, 7]. Thus, a better understanding of the molecular mechanisms regulating tumor progression and immune modulation is essential to identify novel therapeutic targets and improve patient outcomes [8].

**Fig. 1:**
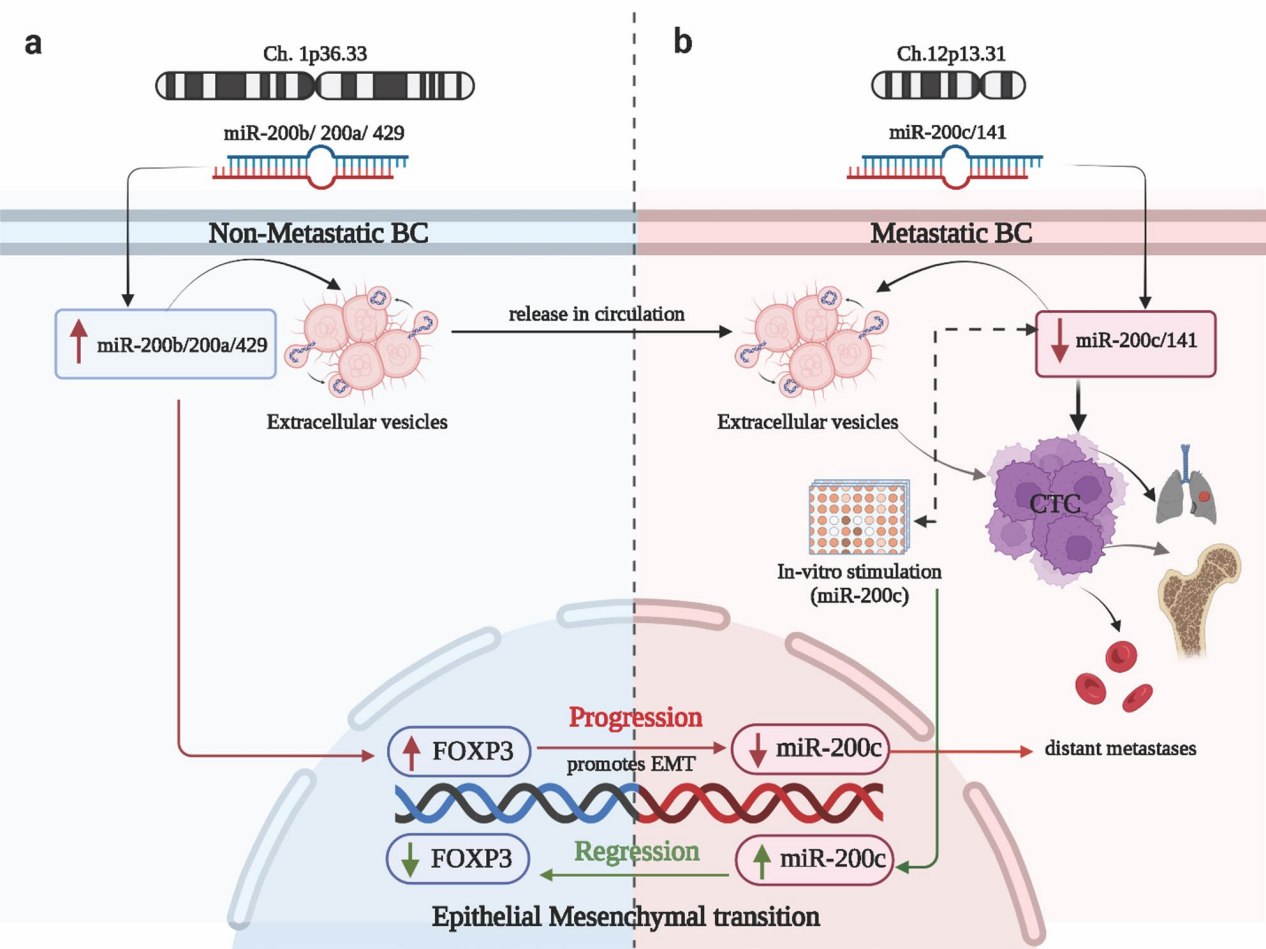
Regulatory networks involving miR-200 family in breast cancer: (**a**) shows the chromosomal locations of the miR-200b/200a/429 cluster, which is located on chromosome 1p36.33. A schematic graph shows high levels of miR-200b/200a/429 in non-metastatic BC, which is attributed to the suppression of FOXP3 and the release of extracellular vesicles (ECVs) derived from tumor cells that are abundant in miR-200 clusters, which further promotes BC progression. **b**: shows the chromosomal locations of miR-200c/141 cluster is located on chromosome 12p13.31. A schematic graph showing low expression of miR-200c/141 in metastatic BC, which subsequently activates FOXP3. In addition, ECVs and CTC are released from tumor cells and localized in distant locations to induce metastases via EMT. Meanwhile, FOXP3 directly activates miR-200c, and in vitro stimulation of miR-200c inhibits EMT, progression, and metastases by reducing the expression of FOXP3. On the other hand, higher levels of miR-200c inhibit FOXP3 “TSG” and promote progression and EMT. Abbreviations: BC: breast cancer, Ch. Chromosome, TSG: tumor suppressor gene, CTC: circulating tumor cells, ECVs: extracellular vesicles, EMT: Epithelial to mesenchymal transition, green arrow: stimulation, red arrow: inhibition

The dysregulation of microRNAs, which are small non-coding RNAs that post-transcriptionally regulate gene expression, has been implicated in breast cancer progression and metastasis [9–12]. Among these microRNAs, miR-200c stands out as a crucial regulator of metastasis by targeting genes involved in the epithelial-mesenchymal transition (EMT) [13, 14]. FOXP3, originally recognised for its function in immune response regulation, has been implicated in the progression of BC and metastases [15]. Recently, a complex interplay between miR-200c and FOXP3 has been identified in regulating BC progression, wherein miR-200c suppresses FOXP3 expression while FOXP3 enhances miR-200c expression [15, 16]. Exploring the miR-200c/FOXP3 interconnection in metastatic breast cancer could unveil novel understandings of the molecular pathways underlying breast cancer progression and metastasis, potentially identifying therapeutic targets [17]. Fig. 1 illustrates the role of the miR-200 family in the progression of breast cancer.

The miR-200c expression has been found to be downregulated in metastatic breast cancer [11], especially lung metastases, in comparison to non-metastatic breast cancer [18, 19]. In many human epithelial malignancies [20, 21], reduced expression of miR-200 family members in metastases compared to primary tumors is frequently linked to poor patient outcomes. Conversely, in mouse models of lung adenocarcinoma and breast cancer, overexpression of miR-200 has been found to inhibit metastasis. There is growing evidence that members of the miR-200 family in circulation can be employed as prognostic/diagnostic biomarkers for cancers, including breast cancer [22], colorectal [23], lung [19], ovarian [24], prostate [25], and bladder [26–28].

FOXP3 is a transcription factor traditionally recognized as the lineage-specifying factor for regulatory T cells (Tregs), which are essential for maintaining immune tolerance and preventing autoimmunity [29]. However, several studies have demonstrated aberrant FOXP3 expression in a variety of epithelial tumors, including breast cancer [30]. Intriguingly, tumor cell-intrinsic FOXP3 has been reported to contribute to immune evasion, chemoresistance, and proliferation, suggesting an oncogenic role in the context of breast cancer [31]. High FOXP3 expression in tumor cells, rather than immune cells, has been correlated with poor prognosis and advanced disease, raising the possibility that FOXP3 may serve as a direct driver of tumor progression [32]. However, the upstream regulators of FOXP3 expression in breast cancer cells remain poorly understood.

Recent computational and experimental analyses have identified FOXP3 as a putative direct target of miR-200c. Bioinformatic predictions using TargetScan and miRanda databases have revealed conserved binding sites for miR-200c within the 3′UTR of FOXP3 mRNA, suggesting the potential for post-transcriptional repression [33]. Furthermore, preliminary studies have shown that restoration of miR-200c expression in breast cancer cell lines results in a significant reduction of FOXP3 protein levels, accompanied by decreased cell viability and migration [15]. These findings point toward a novel regulatory axis in which miR-200c suppresses tumor progression through downregulation of FOXP3, beyond its classical role in EMT inhibition [34, 35].

The therapeutic implications of this axis are considerable. Restoration of miR-200c in breast cancer models has been shown to resensitize tumors to chemotherapy and reduce metastatic potential [36], while inhibition of FOXP3 in tumor cells enhances antitumor immunity and T cell infiltration [37]. These findings suggest that targeting the miR-200c/FOXP3 pathway may offer a dual advantage of direct tumor suppression and immune reactivation. Moreover, miR-200c-based therapeutics are already under preclinical development, with nanoparticle-mediated delivery systems demonstrating effective uptake and functional restoration in vivo [38]. Thus, elucidating the mechanistic role of miR-200c in regulating FOXP3 and tumor progression may contribute to the development of innovative treatment strategies for metastatic and treatment-resistant breast cancer.

Although the MicroRNA Target Prediction Database (miRDB) predicts 279 potential targets for hsa-miR-200c, we selected FOXP3 based on its biological relevance in immune modulation and regulatory T cells within the tumor microenvironment [15]. Additionally, growing evidence from previous research supports a strong link between miR-200c and FOXP3 in regulating epithelial-to-mesenchymal transition (EMT) [21].

The primary objective of this study is to investigate the therapeutic potential of restoring miR-200c expression in metastatic breast cancer and its regulatory impact on FOXP3 protein expression and tumor progression. Specifically, we aim to determine whether systemic administration of a miR-200c mimic can suppress FOXP3 protein levels in tumor cells, thereby inhibiting metastasis and reducing overall tumor burden. Conversely, we assess the pro-tumorigenic effects of miR-200c inhibition, providing mechanistic insights into its loss during disease progression. Through histopathological and molecular analyses, this study seeks to elucidate the functional relationship between miR-200c and FOXP3 in breast cancer and explore the feasibility of miR-200c restoration as a novel therapeutic approach for suppressing tumor progression and immune evasion in metastatic breast cancer.

## Methods

### Cell culture

Three breast cancer cell lines were chosen according to their metastatic status: metastatic BC cells isolated from specific sites [MDA-MB-361 from brain metastasis [39], MDA-MB-468 from pleural effusion [40] of a female with metastatic breast adenocarcinoma, and non-metastatic BC cells MCF7 [41] from the breast tissue of a female patient with ductal carcinoma. The selected BC cell lines reflect three subtypes of BC including MDA-MB-361 (HER2+), which represents HER2-positive BC sensitive to targeted therapy like trastuzumab, MCF-7 (ER+ & PR+), which represents hormonal-dependent breast cancer (BC) sensitive to hormonal therapy, and MDA-MB-468, which represents triple-negative breast cancer (TNBC), which lacks targeted therapies and is frequently treated with chemotherapy. Cells were purchased from the American Type Culture Collection (*Manassas*,* VA*,* USA*). The cell lines were maintained in Ivscove’s modified Dulbecco’s medium (IMDM), supplemented with 1% penicillin /streptomycin and 10% fetal bovine serum (FBS), purchased from *Gibco-BRL*,* Grand Island*,* NY*. Cells were incubated at 37℃ in a humidified incubator with 5% CO2. The media was changed every 48 h until the growth reached 80% confluence, and then the exponentially growing cells were used for all conducted experiments. The cells were passaged every 3 days [42].

### In vitro transfection of miR-200c mimic and inhibitor in human metastatic breast cancer cells

One day before transfection, cells were seeded in a 96-well culture plate. Approximately 1 × 10^5^ cells were seeded in 200 µL of Dulbecco’s Modified Eagle Medium (DMEM), supplemented with10% fetal bovine serum (FBS) and 1% of penicillin G sodium (10.000 UI), streptomycin (10 mg), and amphotericin B (25 µg) (PSA) (*Gibco*,* Thermosientific*,* Germany*). Plates were placed in a 37 °C incubator with 5% CO2 for 24 h to achieve 70% confluence. The following day, a mixture of 0.05µL of miR-200c mimic or 0.5 µL of miR-200c inhibitors in 3 µL of RNase-free water was prepared. 25 µL of the mixture was added into the corresponding well of the 96-well culture plates, providing final concentrations of 5nM and 50nM for miRNA mimic and inhibitor, respectively, after being added to the cells. Next, 0.75 µL of *HI Perfect Transfection Reagent*, *cat no: 301,704*,* Qiagen*,* Hilden*,* Germany*, is added to 24.25 µL of RPMI culture media without serum, and after 10 min incubation at 15–20 °C, 25 µL of the complex is added to each well, followed by the addition of 175 µL of DMEM media. The transfected HNO97 cells were incubated at 37 °C with 5% CO2 for 48 h, after which cell viability was tested via MTT assay.

*AllStars siRNA negative control*,* cat no: 1,027,280*,* Qiagen*,* Hilden*,* Germany*, was used as a negative control for both miRNA mimic and miRNA inhibitor experiments, while *has-miR-1 mimic*,* cat no: MSY0000416*,* Qiagen*,* Hilden*,* Germany*, served as a positive control. Untransfected cells were used for normalization [43].

### Cell proliferation assay

Utilizing the *Vybrant® MTT Cell Proliferation Assay Kit*,* cat. No. M6494*, the cell cytotoxicity assay was carried out (*Thermo Fisher*,* Germany*) after 24 h post-transfection. MiR-200c mimics and inhibitors were transfected into the metastatic breast cancer cells. 100 µL of media was discarded and replaced with new media. At the end of incubation, 20 µL of *4*,*5-dimethylthiazol-2-yl)-2*,*5-diphenyltetrazolium bromide (MTT)* solution (1 mg/mL) from *ThermoScientific*,* Invitrogen*,* Germany* was added to each well. Then, the plates were incubated for four hours at 37 °C with 5% CO2. After that, 100 µL of sodium dodecyl sulfate with hydrochloric acid (SDS-HCL) was added to the wells after the MTT solution had been withdrawn. Using a spectrophotometer, the absorbance at 570 nm was measured to determine the vitality of the cells (*ELx 800; Bio-Tek Instruments Inc.*,* Winooski*,* VT*,* USA*) [44].

### Assessment of apoptosis using Annexin/Propidium iodide staining by flow cytometry

At 48 h after the transfection, the treated cells were collected. *FITC-Annexin V and Propidium iodide (PI)* staining were carried out after trypsinization [45]. Apoptotic cells were separated using the *Dead Cell Apoptosis Kit with Annexin V FITC and PI* for Flow Cytometry (*Invitrogen*,* cat no. V13242)*. The monoclonal antibodies use propidium iodide and recombinant annexin V to detect the externalization of phosphatidylserine in dead cells and apoptotic cells, respectively. Necrotic cells exhibit red fluorescence when stained with propidium iodide. Apoptotic cells exhibit green fluorescence following treatment with both probes, whereas dead cells exhibit red and green fluorescence, and living cells exhibit little or no fluorescence. Data from a flow cytometry experiment were examined using the *Navios software* (*Beckman Coulter*) [46].

### Cell migration and invasion potential by Boyden chamber assay

An in vitro cell migration test was carried out to examine the invasion and migration potential of treated BC cells. Following the transfection for 24 h, the cells were suspended in 200 µL serum-free media and injected using an 8 mm Boyden (ThermoFisher Scientific, Germany) into the upper cavity. As a chemical attractant, a 10% fetal bovine serum-containing medium was introduced to the lumen. They were incubated for 24 h at 37 °C. An inverted microscope was used to obtain pictures after the lower surface cells were preserved with methanol and the upper lumen cells were removed using cotton swabs. The number of migrated cells was quantified by fixing and crystal violet–staining the invaded cells on the lower surface of the Matrigel-coated membrane, after removal of non-migrated cells. Images were captured using an inverted microscope, and cell quantification was performed using ImageJ software (NIH, USA) with the Cell Counter plugin, by counting cells in five randomly selected microscopic fields (20× magnification) per insert results were expressed as the mean ± SD of migrated cells for each group [47].

### MiR-200 and FOXP3 gene expression using real-time PCR

An average of 1 × 10^6^ cells underwent disruption and homogenization through bead-milling in a lysis buffer containing guanidine-thiocyanate for total RNA extraction. The Tissue Ruptor II was utilized to disrupt and homogenize the cells further. The resulting mixture underwent centrifugation, and the cell supernatant was collected for RNA extraction. Total RNA extraction from cells and tumor xenograft tissue was carried out using the miRNeasy Mini kit, cat no: 217,084 (*Qiagen*,* Hilden*,* Germany*). Reverse transcription was performed using the QuantiTect Reverse Transcription Kit and miRCURY LNA RT Kit. cDNA synthesis involved incubation of the reaction mix at 42 °C for 60 min, followed by enzyme inactivation at 95 °C for 5 min and cooling at 4 °C. Amplification of miR-200c gene expression levels from cDNA was done using the miRCURY LNA PCR Assay, hsa-miR-200c-5p, cat.no: 339,306, assay ID: YP02119294, sequence: 5’CGUCUUACCCAGCAGUGUUUGG, (Qiagen, Hilden, Germany). FOXP3 gene amplification in BC cells and xenograft tumor tissue was performed using the Hs_ FOX3 QuantiTect Primer Assay, cat. No. 249,900, assay ID: QT00048286, and the Mm_Foxp3 QuantiTect primer assay, assay ID: QT00138369 (Qiagen, Germany), respectively. Real-time PCR was conducted using the QuantiTect SYBR Green PCR Kit. Expression levels were standardized using β-actin as a reference gene and analyzed using the 2-∆∆Ct equation [48].

### Metastatic breast cancer induction and treatment

All animal experiments were conducted in compliance with institutional ethical guidelines and approved by the *Institutional Animal Care and Use Committee* (IACUC). Female athymic nude mice (6–8 weeks old, 20–22 g); obtained from *Animal House*,* Ain Shams Medical Research Center*,* Faculty of Medicine*,* Ain Shams University*,* Cairo*, *Egypt* were randomly divided into five groups (*n* = 6 per group): (1) Healthy control; (2) Metastatic BC model; (3) Metastatic BC + miR-200c mimic; (4) Metastatic BC + miR-200c inhibitor; and (5) Metastatic BC + Cisplatin. To establish the metastatic breast cancer model, mice in groups 2–5 were anesthetized with isoflurane and intracardially injected with 1 × 10⁵ MDA-MB-361 cells (ATCC- HTB-27, derived from brain metastasis) and 1 × 10⁵ MDA-MB-468 cells (ATCC- HTB-132, derived from pleural effusion of a female with metastatic adenocarcinoma) in 100 µL sterile PBS per mouse, using a 27G insulin syringe under ultrasound guidance, as previously described [49]. Cells were cultured in DMEM (Gibco, Cat. No. 11965092) supplemented with 10% FBS (Gibco, Cat. No. 26140079) and 1% penicillin–streptomycin (Gibco, Cat. No. 15140122). Group 3 received tail vein injections of miR-200c mimic (10 nmol/mouse, Ambion, Thermo Fisher, Cat. No. 4464066, Assay ID: MC10761) complexed with Invivofectamine 3.0 reagent (Thermo Fisher Scientific, Cat. No. IVF3005) every 3 days for 3 weeks. Group 4 received miR-200c inhibitor (10 nmol/mouse, Ambion, Cat. No. 4464084, Assay ID: MH10761) delivered similarly. Group 5 received Cisplatin intraperitoneally (5 mg/kg, Sigma-Aldrich, Cat. No. P4394) twice weekly as a positive control. Healthy controls received vehicle-only Phosphate buffer saline (PBS). Mice were monitored daily for signs of distress and weight loss as an indication of metastasis. At the end of the 4-week period, animals were euthanized by CO₂ inhalation. Brain, lung, and liver tissues were harvested and fixed in 10% neutral-buffered formalin for histological analysis, while additional tissue aliquots were snap-frozen in liquid nitrogen and stored at − 80 °C for molecular analyses, including RNA extraction and qRT-PCR.

### Immunohistochemical analysis

The anti-mouse FOXP3 monoclonal antibody, cat no: 14-4774 (*eBioscience*,* Invitrogen*,* USA*) was used to analyze FOXP3 expression via immunohistochemistry. Paraffin-embedded tissue samples were sectioned into 4-µm thicknesses and placed on a coated glass slide. To block intrinsic peroxidase activity, specimens were treated with a peroxidase-blocking reagent (*Dako Cytomation*,* Glostrup*,* Denmark*) for 5 min. Antigens were recovered by boiling the specimens in 1mmol/L l̸ EDTA (pH 9.0) target retrieval solution (*Dako Cytomation*) for 30 min in a microwave. After washing in Tris-buffered saline (*TBS; Dako Cytomation*) for 10 min, the specimens were incubated with a 1:400 dilution of the FOXP3 antibody. The histological samples were then incubated overnight at 4˚C, followed by a 15-minute wash with TBS, and then incubated with a labeled polymer-horseradish peroxidase (HRP) secondary antibody (*ChemMate Envision kit; Dako Cytomation)* for 30 min at room temperature. After washing in TBS for 10 min, the slides were visualized using 3,3’-diaminobenzidine. Two blinded pathologists independently assessed the expression of FOXP3 without knowledge of the experimental groups [50]. The staining intensity of FOXP3‑positivity (FOXP3+) within the tumor-cell cytoplasm was recorded. FOXP3-positivity staining intensity within the cytoplasm was classified as either weak (1+), moderate (2+), or strong (3+). The number of FOXP3 + cells was manually counted in 10 different fields at 400x magnification.

### Statistical analysis

The data were reported as the mean ± standard deviation (SD). Statistical analysis was performed using GraphPad Prism version 9.0.0 for Windows, "http://www.graphpad.com" (*GraphPad Software*,* San Diego*,* California*,* USA*). Group comparisons were conducted using Student’s t-test, Fisher’s exact test, and one-way ANOVA followed by Dunnett’s or Tukey’s multiple comparisons test according to the distribution of data. Pearson’s correlation was employed to assess the strength of the linear relationship between two variables. A significance level of *p* < 0.05 was considered statistically significant.

## Results

### MiR-200c restoration suppresses proliferation in metastatic breast cancer cells

**Fig. 2:**
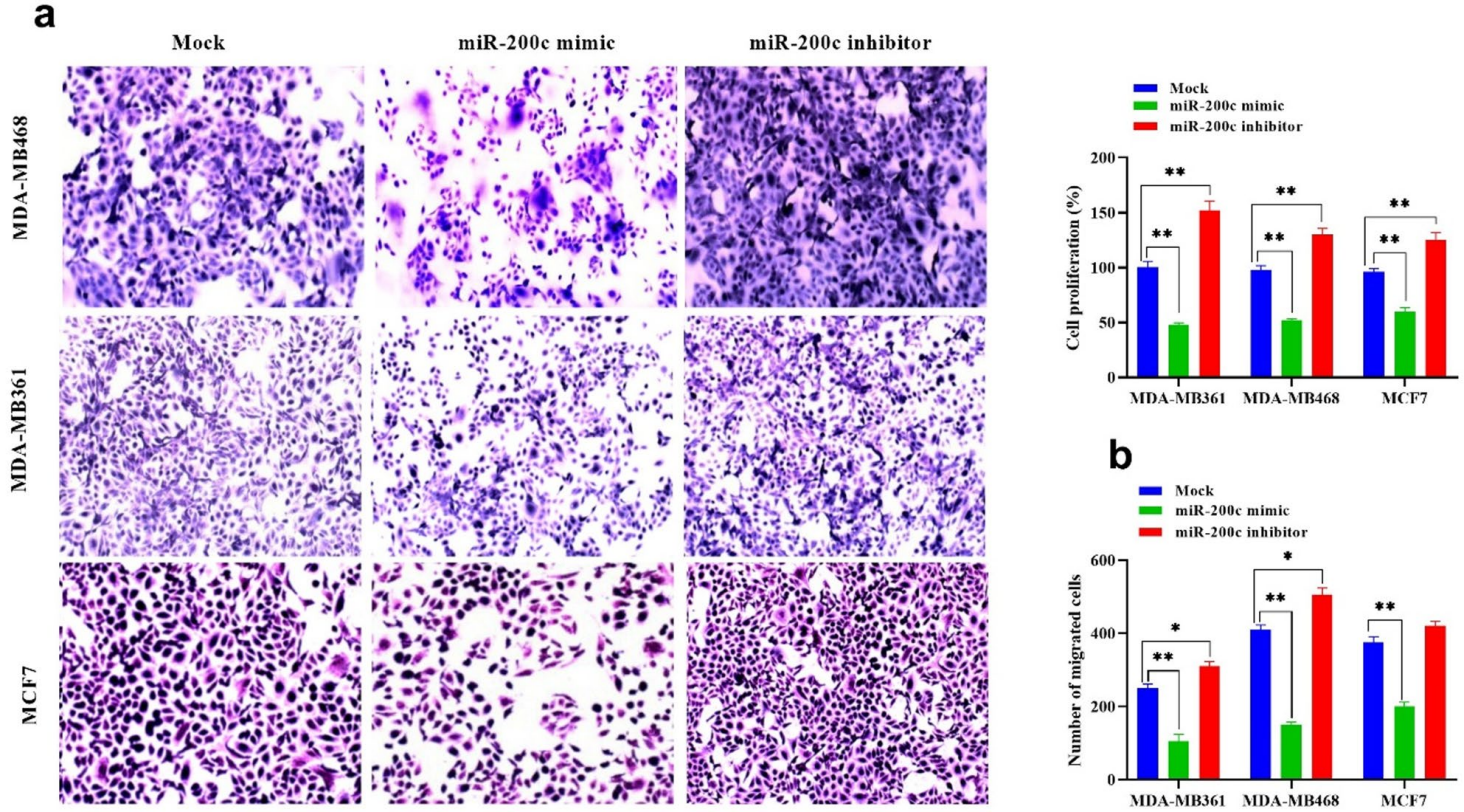
Restoration of miR200c inhibits non-metastatic and metastatic breast cancer cell proliferation and migration. **a** Effect of miR-200c modulation on viability of breast cancer cells, (**b**) Light microscope images of migrated cells, “cell migration assay,” stained with crystal violet, showed a significant reduction in the migration in BC cells transfected with miR-200c mimic. However, a significant increase in migrated cells was detected in BC cells after transfection with miR-200c inhibitor. **c** Effects of transfection of BC cells with miR-200c mimic and inhibitor on cell invasion measured by Boyden chamber invasion assay. The sample is performed in triplicate to ensure the accuracy of the test. Values represent the mean of three experiments ± Standard deviation, **, p-value < 0.01, *, p-value < 0.05. Comparative analysis was performed by One-Way ANOVA, followed by Tukey’s multicomparative test. Abbreviations: BC: breast cancer

To investigate the functional impact of miR-200c on breast cancer cell viability and invasiveness, metastatic (MDA-MB-361 and MDA-MB-468) and non-metastatic (MCF-7) breast cancer cell lines were transfected with a synthetic miR-200c mimic or inhibitor. MTT assay results revealed a significant reduction in cell viability following treatment with miR-200c mimic, particularly in the metastatic MDA-MB-361 and MDA-MB-468 cells, compared to the non-metastatic MCF-7 line. The cytotoxic effect of miR-200c restoration was more pronounced in metastatic cells (Fig. 2a).

### Effect of miR-200c modulation on cell invasion in breast cancer cell lines

To determine the role of miR-200c in regulating breast cancer cell invasiveness, a Boyden chamber invasion assay was performed in MDA-MB361, MDA-MB468, and MCF7 cells following transfection with either a miR-200c mimic or inhibitor (Fig. 2b and c). In MDA-MB361 cells, the number of migrated cells in the untreated control group was 250 ± 10.6, which was significantly reduced to 105 ± 18.3 upon miR-200c mimic transfection (*p* < 0.01), representing an approximate 58% decrease. Conversely, inhibition of miR-200c increased migration to 310 ± 12.6 cells (*p* < 0.05), a 24% increase compared with control. In MDA-MB468 cells, which displayed the highest baseline invasion (410 ± 12.6 migrated cells), miR-200c mimic expression resulted in a marked suppression to 150 ± 7.5 cells (*p* < 0.001; 63% reduction), whereas inhibition enhanced migration to 505 ± 18.5 cells (*p* < 0.01; 23% increase). In MCF7 cells, untreated controls exhibited 375 ± 15.8 migrated cells, which declined significantly to 200 ± 11.6 following mimic transfection (*p* < 0.01; 47% reduction), while inhibition led to a moderate increase to 420 ± 12.8 cells (*p* < 0.05; 12% increase).

### MiR-200c stimulation triggers apoptosis in metastatic breast cancer cells

**Fig. 3:**
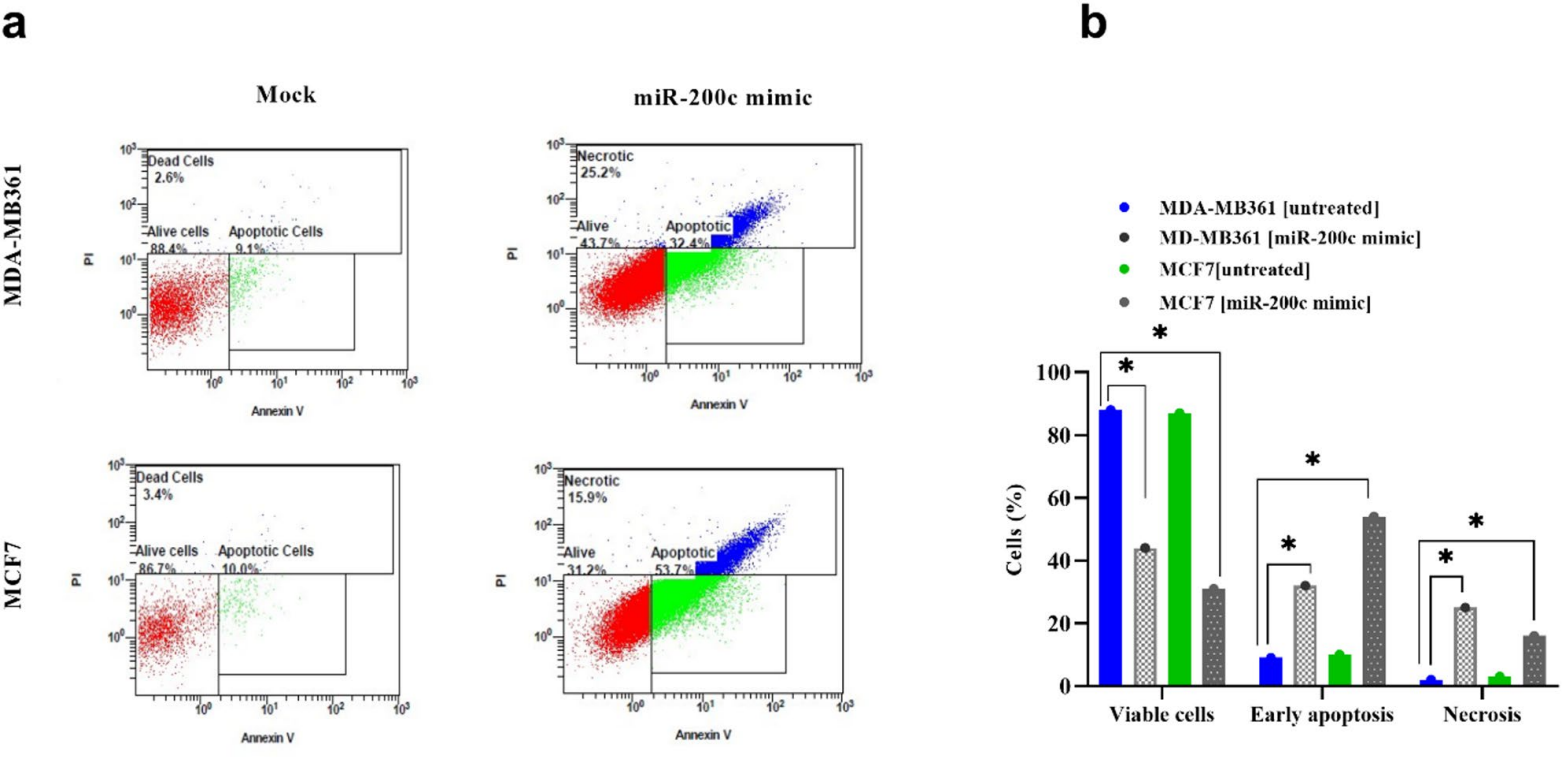
Effect of transfection with miR-200c mimic on apoptosis of MDA-MB361 and MCF7 cell lines (**a**) Images of apoptosis assay by Annexin V/propidium iodide following transfection of miR-200c mimic, measured by Flowcytometry. **b** Percent of viable, early apoptotic, and necrotic cells. Values represent the mean of three experiments ± Standard deviation of three independent measurements for each group. * indicates significant difference between compared groups (*p* < 0.05).Multiple group cmparisions was performed by One-Way ANOVA, followed by Tukey’s multicomparative test. Abbreviations: PI: propidium iodide, BC: breast cancer, PI: Propidium iodide

Since miR-200c expression was downregulated in breast cancer cell lines, we next investigated how Annexin V/PI assays would respond if the miR-200c expression was restored through transfection of miR-200c mimic. We chose to carry out these studies using the non-invasive MCF-7 and the invasive MDA-MB-361 cell lines, considering the results. The results show that transfection with miR-200c mimic enhances apoptosis in MCF7 and MDA-MB361 cells, respectively (Fig. 3a). A high significant difference was detected regarding the % of apoptotic cells between invasive MDA-MB361 and non-invasive MCF7 cells after transfection with miR-200c mimic (mean difference: -22.0, 95% CI: -27.95 to -16.05, *p* < 0.001). On the other hand, a marked increase in the percentage of necrotic cells was significantly associated with MDA-MB361 cells transfected with miR-200c mimic, compared to MCF-7 transfected cells (mean difference: 9.30, 95%CI: 3.56 to 15.04, *p* = 0.004) (Fig. 3b).

### MiR-200c modulates FOXP3 expression in metastatic breast cancer cells

**Fig. 4:**
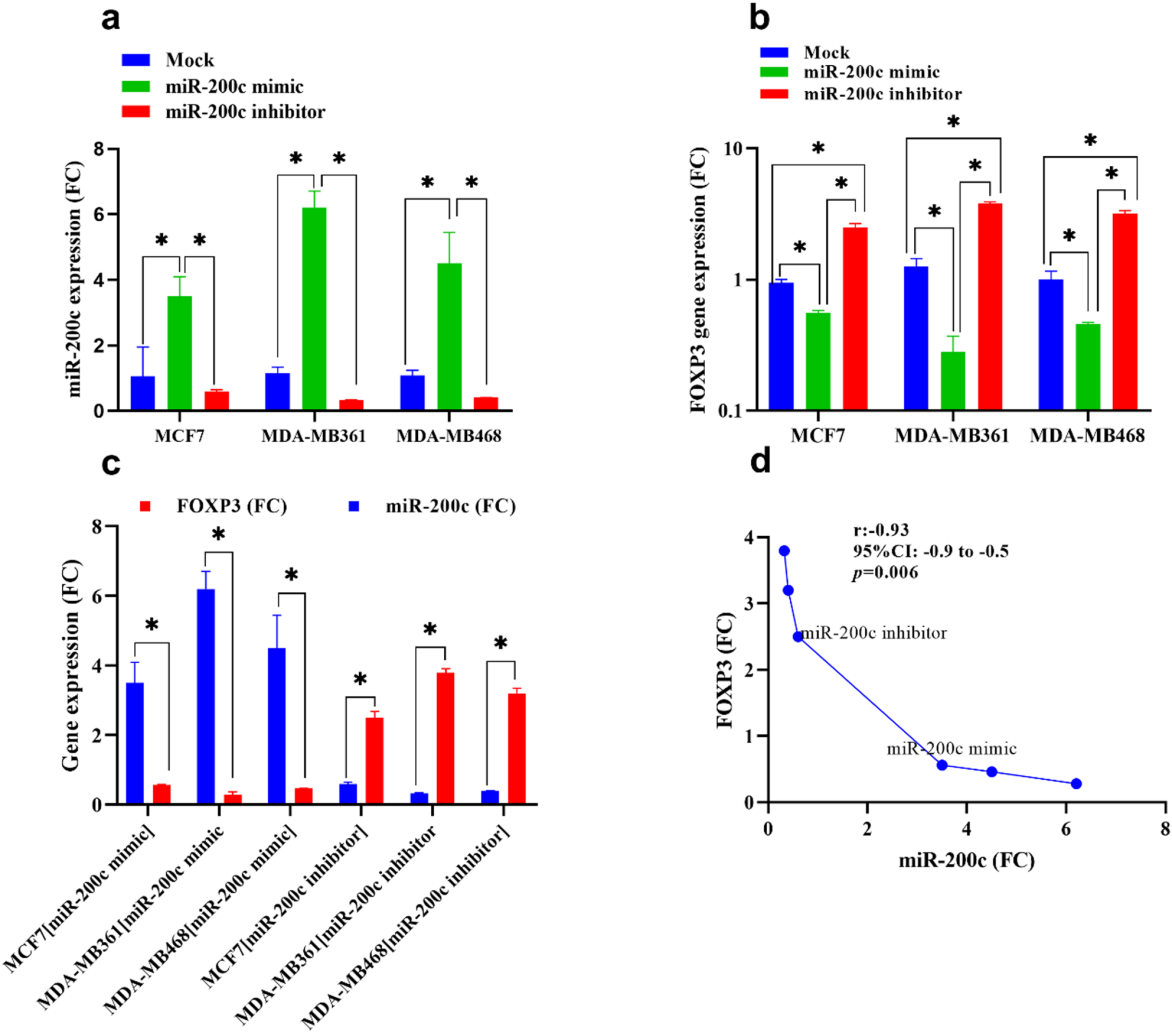
Effect of transfection with miR-200c mimic on the expression of miR-200c-5p and its target gene “mRNA-FOXP3”, measured by Syber green-based Real-time PCR. Values represent the mean of three experiments ± Standard deviation in three tested BC cell lines. **a** and **b**: miR-200c-5p and mRNA-FOXP3 expression (FC) after transfection with miR-200c mimic or inhibitor, compared to untreated cells. Values represent the mean of three independent measurements for each experiment ± Standard deviation. *: denotes a significant difference between the compared groups (*p* < 0.05). Statistical significance was determined using a one-way ANOVA followed by Tukey’s post-hoc test. **c** Pearson’s correlation between the expression of miR-200c and FOXP3 in different groups. Abbreviations: BC: breast cancer, FC: fold change, r: correlation coefficient, CI: confidence interval

To evaluate the regulatory relationship between miR-200c and FOXP3 expression in breast cancer, we performed quantitative real-time PCR (qRT-PCR) analysis in both metastatic (MDA-MB-361 and MDA-MB-468) and non-metastatic (MCF-7) breast cancer cell lines following transfection with either a miR-200c mimic or inhibitor. As shown in Fig. 4a, transfection with the miR-200c mimic resulted in a significant upregulation of miR-200c-5p expression in all tested cell lines compared to untreated controls (*p* < 0.05). This increase in miR-200c expression was accompanied by a downregulation of FOXP3 mRNA levels, with the most pronounced suppression observed in MDA-MB-361 cells (Figs. 4b and c).

Conversely, inhibition of miR-200c led to a significant increase in FOXP3 transcript levels in both metastatic and non-metastatic cells (*p* < 0.05). These findings suggest that miR-200c negatively regulates FOXP3 at the mRNA level.

To further investigate the relationship between miR-200c and FOXP3, a Pearson correlation analysis was conducted. The analysis revealed a strong and statistically significant inverse correlation between miR-200c-5p and FOXP3 expression across the examined breast cancer cell lines (*r* < 0, *p* < 0.01), as depicted in Fig. 4d. Together, these results provide compelling evidence that miR-200c directly or indirectly represses FOXP3 gene expression, potentially contributing to its tumor-suppressive effects in breast cancer.

### Restoration of miR-200c reduces metastatic tumor burden and FOXP3 expression in vivo

To assess the therapeutic efficacy of miR-200c restoration in vivo, we examined metastatic burden and FOXP3 expression in brain, lung, and breast tissues collected from mice injected with MDA-MB-361 and MDA-MB-468 cells.

**Fig. 5:**
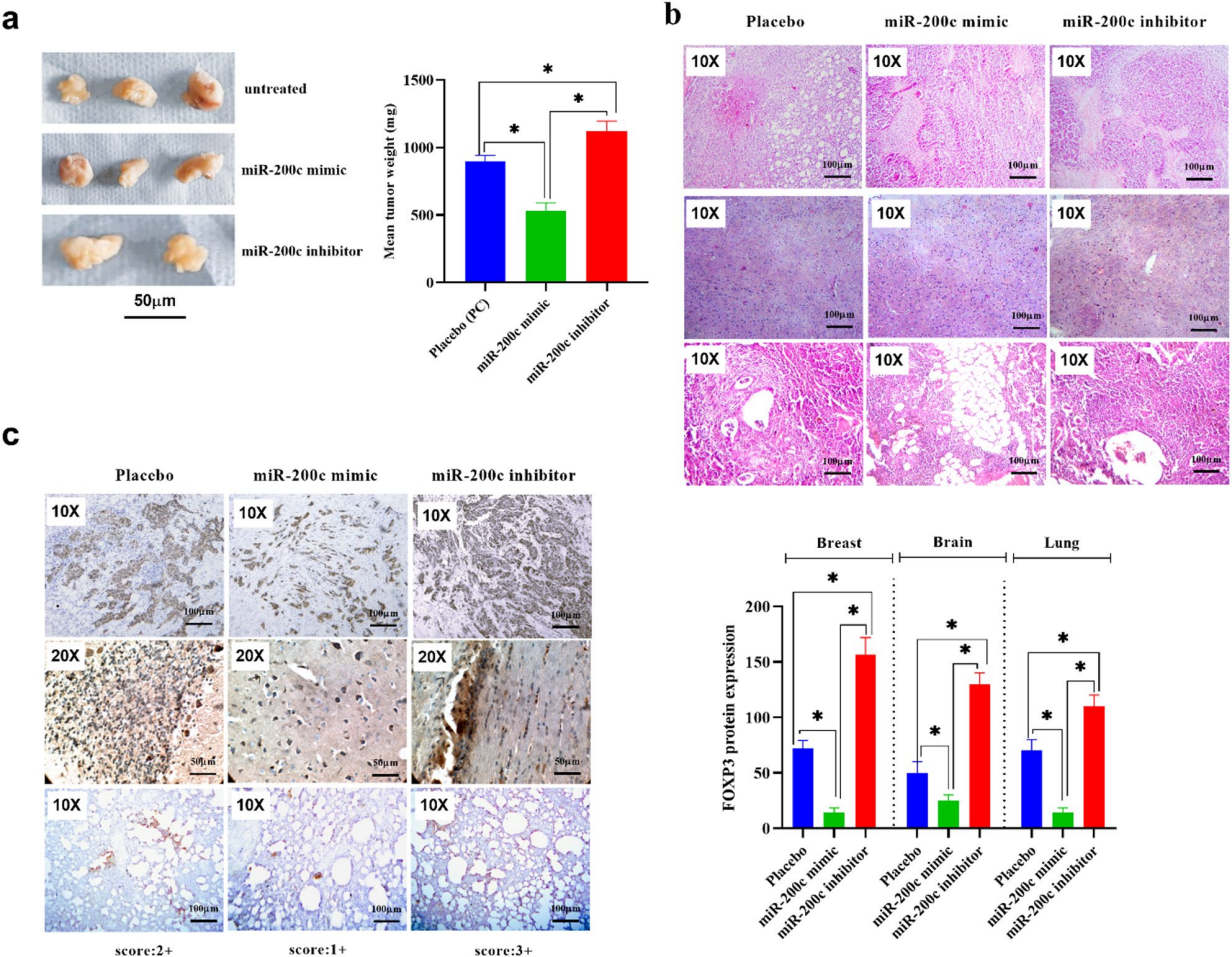
Restoration of miR-200c decreases breast cancer xenograft tumor growth. **a**: present the tumor weight measured in tumors transfected with miR-200c mimic or inhibitors, compared to untreated tumor tissue. **b**: displays images stained with Hematoxylin and Eosin of breast cancer at both magnifications x100 and x400, along with breast cancer tumor size and immunohistochemical staining of forkhead box protein 3 (FOXP3) in breast cancer. **c**: FOXP3 expression is illustrated in representative images with nuclear and cytoplasmic localization at magnification x100, with the immunoreactive score (IRS) of each group presented on the respective image. Values represent the mean of three independent measurements for each experiment ± Standard deviation.*: denotes significance between the compared groups (*p* < 0.05). Statistical analysis was performed using one-way ANOVA followed by Tukey’s post-hoc test to determine differences among groups. Abbreviations: H&E: Hematoxylin and eosin, FOXP3: Forkhead box protein 3, IRS: immunoreactive score

A significant reduction in tumor weight was detected in tumor tissue exposed to miR-200c mimic, compared to untreated tumor tissue (mean difference: 359, 95%CI: 216.3 to 503.0, *p* = 0.001). Meanwhile, tumor tissue exposed to miR-200c inhibitor exhibits a marked increase in tumor weight (mean difference: -226, 95%CI: -369 to 82.67, *p* = 0.007). The decrease in breast cancer tumor weight observed in tumors exposed to the miR-200c mimic and the increase in tumor weight in tumors exposed to the miR-200c inhibitor represent contrasting effects of modulating miR-200c expression on breast cancer growth (Fig. 5a).

#### Histological findings

Hematoxylin and eosin (H&E) staining revealed extensive metastatic infiltration in the brain and lung tissues of untreated and miR-200c inhibitor–treated groups, characterized by dense tumor cell clusters, disrupted parenchymal architecture, and evidence of perivascular invasion. In contrast, mice treated with the miR-200c mimic exhibited significantly fewer metastatic lesions, with preserved tissue structure and sparse micrometastatic foci, particularly in the brain (*p* < 0.01). Lung metastases were similarly reduced in the mimic-treated group, while cisplatin-treated mice showed moderate suppression of metastatic spread compared to the untreated controls (Fig. 5b).

#### FOXP3 orotein expression

Immunohistochemical (IHC) staining using anti-FOXP3 antibody further supported these findings. Strong nuclear and cytoplasmic FOXP3 immunoreactivity was observed in tumor cells within metastatic lesions of the untreated (IRS: +2) and miR-200c inhibitor–treated mice (IRS: +3), while FOXP3 expression was markedly attenuated in the miR-200c mimic group (IRS: +1). Quantitative analysis of FOXP3-positive cells showed a significant reduction in IHC scores in brain and lung tissues of mimic-treated animals (*p* < 0.01), confirming that miR-200c negatively regulates FOXP3 protein expression in vivo (Fig. 5c).

### Restoration of miR-200c inhibits the FOXP3 gene expression

**Fig. 6:**
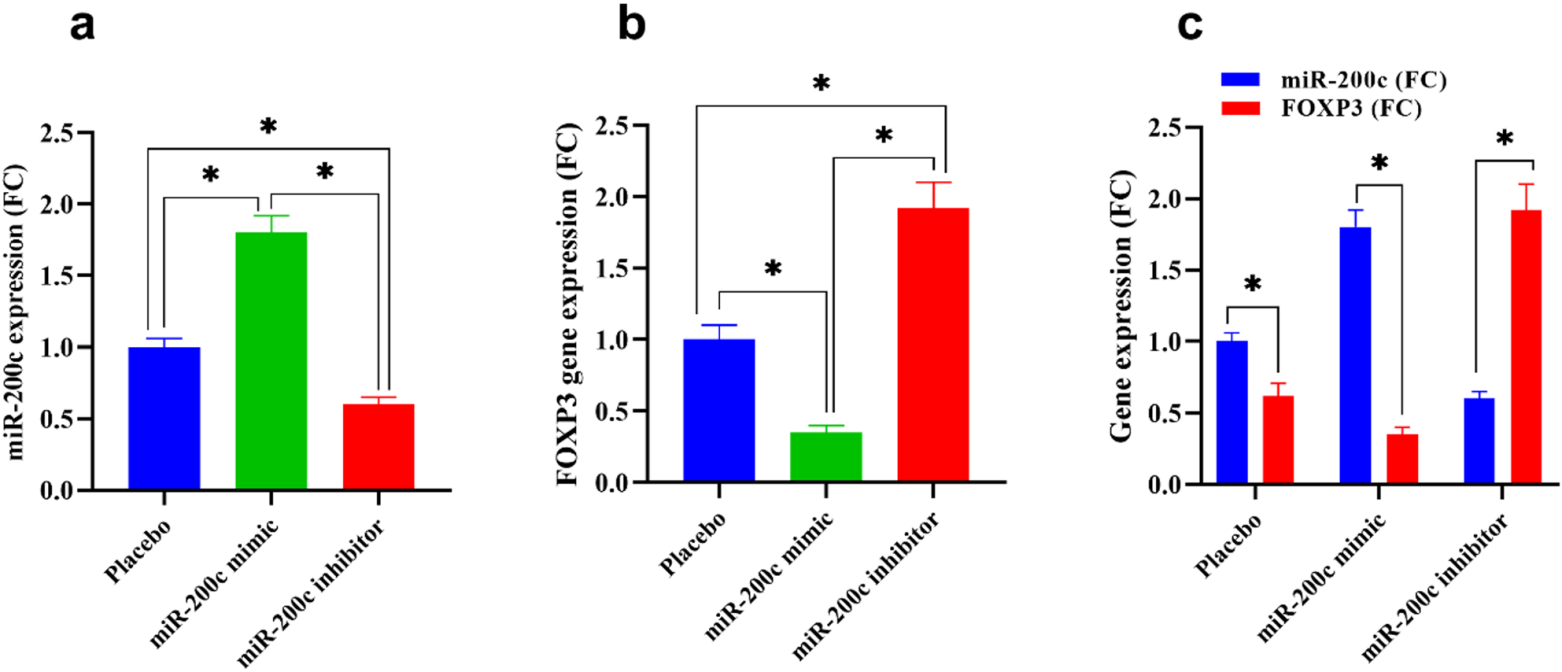
Restoration of miR-200c inhibits FOXP3 gene expression in breast cancer xenograft tumors. The expression level of miR-200c-5p (**a**) and FOXP3 (**b**) in tumor tissue was measured by RT-PCR. **c**: association between miR-200c-5p and mRNA-FOXP3 gene expression in tumor tissue. Values represent the mean of three experiments ± Standard deviation. Statistical analysis was performed using one-way ANOVA followed by Tukey’s multiple comparisons test.*: Denotes significant difference between compared groups (*p* < 0.05)

At the molecular level, qRT-PCR analysis of dissected tissues demonstrated robust overexpression of miR-200c in the mimic-treated group relative to controls (*p* < 0.001) (Fig. 6a), accompanied by significant downregulation of FOXP3 mRNA (*p* < 0.01) (Fig. 6b). Conversely, animals treated with the miR-200c inhibitor showed suppressed miR-200c levels and elevated FOXP3 gene expression in all analyzed tissues. Notably, Pearson correlation analysis across samples revealed a strong inverse correlation between miR-200c and FOXP3 transcript levels (*r* = − 0.82, *p* < 0.01), mirroring in vitro findings (Fig. 6c**)**.

## Discussion

Metastatic breast cancer remains a major clinical challenge due to its aggressive nature, limited therapeutic options, and poor prognosis [51]. In this study, we investigated the therapeutic potential of modulating miR-200c levels to regulate the expression of FOXP3, a transcription factor increasingly implicated in tumor progression and immune evasion. Our findings demonstrate that restoration of miR-200c suppresses tumor proliferation, invasion, and FOXP3 expression both in vitro and in vivo, thus highlighting the miR-200c/FOXP3 axis as a promising target in metastatic breast cancer treatment.

miR-200c is known to act as a tumor suppressor by targeting transcriptional regulators involved in epithelial–mesenchymal transition (EMT) [52], FOXP3, a transcription factor with context-dependent oncogenic activity in breast cancer, may be downregulated by miR-200c through post-transcriptional repression of its mRNA 3′-UTR. This interaction likely disrupts the FOXP3-mediated transcriptional network that promotes invasion and metastasis, thereby contributing to the anti-metastatic effects observed upon miR-200c restoration [21]. Consistent with previous studies (Gregory et al., 2008; Korpal et al., 2008), our data show that restoring miR-200c expression significantly inhibits the viability and invasive potential of metastatic breast cancer cells (MDA-MB-361 and MDA-MB-468), while exerting a milder effect on non-metastatic MCF-7 cells [53, 54]. The results from the MTT and transwell invasion assays clearly illustrate this selective sensitivity, suggesting that miR-200c targets molecular pathways that are particularly active in aggressive breast cancer phenotypes.

In addition to its effects on proliferation and migration, miR-200c overexpression also triggered apoptosis, particularly in metastatic MDA-MB-361 cells. The increased proportion of Annexin V-positive apoptotic cells and necrotic cells following transfection of miR-200c mimic indicates a pro-death effect, which may contribute to its tumor-suppressive activity. This aligns with earlier findings showing that miR-200c modulates apoptotic regulators and sensitizes cancer cells to chemotherapeutic agents [55, 56]. The stronger pro-apoptotic effect observed in MDA-MB-361 compared to MCF-7 further underscores the differential response of metastatic versus non-metastatic cells to miR-200c modulation.

Previous research suggests that miR-200c may suppress tumor growth. Li et al. [57] found a downregulation of miR-200c expression in BC tumor tissues and cell lines [58]. Additionally, Song et al. [43] and Zuberi et al. [59] observed miR-200c downregulation across eight different BC cell lines and malignancies. Li et al. further investigated miR-34a and miR-200c expression patterns, revealing that co-transfection of these miR-34a and miR-200c could promote apoptosis and G2/M cell cycle arrest while suppressing malignant cellular features crucial for the growth of breast cancer, including proliferation, invasion, migration, and the expression of significant markers of EMT and stemness.

Recent studies have underscored the clinical significance of circulating miR-200 in BC patients, associating its levels with poor prognosis [11], metastasis occurring up to two years prior to clinical diagnosis, and disease progression [16]. Moreover, patients with metastatic BC in a previous trial exhibited notably higher plasma levels of miR-200c compared to those with localized BC at the time of diagnosis [11].

In many cellular contexts, functional studies have shown contradictory findings regarding the function of miR-200 in suppressing or promoting metastasis [60, 61]. Similarly, recent research suggested that miR-200c and miR-141 had higher expression levels in metastatic breast tumors [62], while more recent research showed that miR-200c expression was lower in primary breast tumors with lymph nodes [63]. MiR-200 s was found to be downregulated in primary breast cancer cells in patients with distant metastases by analysis of the TCGA database.

Importantly, our study strongly suggest, though do not directly confirm, that miR-200c directly influences the expression of FOXP3, a transcription factor best known for its role in regulatory T cells (Tregs) but increasingly recognized for its aberrant expression in tumor cells. Through qRT-PCR analyses in breast cancer cell lines, we demonstrate that miR-200c mimic treatment leads to significant downregulation of FOXP3 mRNA, whereas inhibition of miR-200c results in its upregulation. These findings are supported by a strong negative correlation between miR-200c and FOXP3 levels, suggesting an inverse regulatory relationship. This novel insight into the post-transcriptional regulation of FOXP3 by miR-200c adds to our understanding of how non-coding RNAs shape the oncogenic landscape.

FOXP3 has traditionally been viewed as a tumor suppressor gene in breast cancer [31, 64]; however, accumulating evidence suggests that its role is context-dependent. In some settings, FOXP3 expression in tumor cells has been linked to enhanced proliferation, immune evasion, and resistance to therapy [65]. Our results are in line with this oncogenic paradigm: metastatic breast cancer cells expressing high levels of FOXP3 were more aggressive, and suppression of FOXP3 by miR-200c was associated with reduced tumor burden and increased apoptosis. This shift in understanding positions FOXP3 not only as an immune regulator but also as a functional promoter of metastatic traits in tumor cells [66]. In addition, MiR-200c and miR-141 have been found to be downregulated in primary breast cancer cells using the *Foxp3* ^sf/-^deficient spontaneous breast cancer mouse model [67], particularly in animals with lung metastases. Accordingly, these findings imply that miR-200c/141, which is activated by FOXP3, may act as metastatic suppressors at original sites but as metastatic promoters at metastatic sites [15].

The in vivo data further reinforce the therapeutic relevance of miR-200c. In our orthotopic model of metastatic breast cancer, animals treated with miR-200c mimic showed significantly reduced tumor weight, metastatic burden, and FOXP3 expression in the brain, lungs, and mammary tissues. H&E staining confirmed fewer and smaller metastatic foci, while IHC analysis revealed a marked reduction in FOXP3 protein expression in the mimic-treated group. These findings not only validate the in vitro observations but also demonstrate the feasibility of miR-200c–based interventions in a preclinical setting. The contrast between the miR-200c mimic and inhibitor groups, particularly regarding FOXP3 levels and tumor aggressiveness, strongly supports a causal relationship between miR-200c modulation and FOXP3-mediated tumor behavior.

Moreover, the consistent downregulation of FOXP3 at both mRNA and protein levels in miR-200c–treated tissues underscores the robustness of this regulatory axis. The inverse correlation between miR-200c and FOXP3 observed across all experimental conditions, including correlation analyses, adds mechanistic credibility to the notion that miR-200c acts as a post-transcriptional suppressor of FOXP3 [68]. This relationship may be direct via binding to the 3’UTR of FOXP3 mRNA or indirect, through intermediary regulatory pathways [15], which warrants further investigation using luciferase reporter assays and miRNA pull-down techniques.

From a therapeutic perspective, the findings of this study suggest that restoring miR-200c expression in metastatic breast cancer may offer dual benefits: direct suppression of tumor growth and indirect modulation of the tumor microenvironment via FOXP3 downregulation. Given the growing interest in combining immunomodulatory strategies with molecular therapies, targeting the miR-200c/FOXP3 axis could enhance antitumor immune responses by reducing FOXP3-mediated immunosuppression in the tumor niche. This strategy may also complement conventional therapies such as chemotherapy, as indicated by the comparable effects observed in the cisplatin-treated group.

While our findings are promising, several limitations should be acknowledged. First, the study focuses on two metastatic breast cancer cell lines and a single miRNA–gene axis. Broader validation across different subtypes would strengthen the generalizability of our conclusions. Second, although correlation analyses suggest regulatory interactions, definitive proof of direct miR-200c targeting of FOXP3 requires additional functional assays, such as 3′UTR luciferase reporters and site-directed mutagenesis.

## Conclusions

In conclusion, this study identifies miR-200c as a potent suppressor of breast cancer metastasis through the downregulation of FOXP3 expression. The restoration of miR-200c not only impairs tumor proliferation and invasion but also attenuates FOXP3-driven oncogenic signaling, suggesting a novel therapeutic axis with significant translational potential. These findings provide a strong rationale for the continued exploration of miR-200c–based therapies and underscore the importance of non-coding RNA–mediated gene regulation in the progression and treatment of metastatic breast cancer.

## Data Availability

The datasets used and/or analyzed during the current study are available from the corresponding author upon reasonable request.

## Abbreviations

miR-200c: MicroRNA-200c
EMT: Epithelial-Mesenchymal Transition
Tregs: Regulatory T Cells
miRDB: MicroRNA Target Prediction Database
ECVs: Extracellular Vesicles
BC: Breast Cancer
TNBC: Triple-Negative Breast Cancer
IMDM: Ivscove’s Modified Dulbecco’s Medium
FBS: Fetal Bovine Serum
DMEM: Dulbecco’s Modified Eagle Medium
SDS-HCL: Sodium Dodecyl Sulfate with Hydrochloric Acid
PI: Propidium Iodide
IACUC: Institutional Animal Care and Use Committee
PBS: Phosphate Buffer Saline
TBS: Tris-Buffered Saline
HRP: Horseradish Peroxidase
SD: Standard Deviation
qRT-PCR: Quantitative Real-Time PCR
H&E: Hematoxylin and Eosin
IHC: Immunohistochemical
IRS: Immunoreactive Score
